# Modulation of miRNAs in Pulmonary Hypertension

**DOI:** 10.1155/2015/169069

**Published:** 2015-03-11

**Authors:** Sudhiranjan Gupta, Li Li

**Affiliations:** ^1^Division of Molecular Cardiology, Department of Medicine, Texas A&M Health Science Center College of Medicine, Temple, TX 76504, USA; ^2^Baylor Scott & White Health, Temple, TX 76508, USA; ^3^Central Texas Veterans Health Care System, Temple, TX 76504, USA; ^4^Department of Physiology and Pathophysiology, Peking University Health Science Center, Beijing 100191, China

## Abstract

MicroRNAs (miRNAs) have emerged as a new class of posttranscriptional regulators of many cardiac and vascular diseases. They are a class of small, noncoding RNAs that contributes crucial roles typically through binding of the 3′-untranslated region of mRNA. A single miRNA may influence several signaling pathways associated with cardiac remodeling by targeting multiple genes. Pulmonary hypertension (PH) is a rare disorder characterized by progressive obliteration of pulmonary (micro) vasculature that results in elevated vascular resistance, leading to right ventricular hypertrophy (RVH) and RV failure. The pathology of PH involves vascular cell remodeling including pulmonary arterial endothelial cell (PAEC) dysfunction and pulmonary arterial smooth muscle cell (PASMC) proliferation. There is no cure for this disease. Thus, novel intervention pathways that govern PH induced RVH may result in new treatment modalities. Current therapies are limited to reverse the vascular remodeling. Recent studies have demonstrated the roles of various miRNAs in the pathogenesis of PH and pulmonary disorders. This review provides an overview of recent discoveries on the role of miRNAs in the pathogenesis of PH and discusses the potential for miRNAs as therapeutic targets and biomarkers of PH at clinical setting.

## 1. Introduction

Pulmonary hypertension (PH) is predominantly defined by a mean pulmonary artery pressure at rest greater than or equal to 25 mm Hg. It is an enigmatic vascular disease and the pathogenesis of PH is multifactorial of origin and, hence, is categorized as idiopathic type [1–4]. As PH develops in a wide variety of clinical circumstances and is associated with diverse histological manifestations, a classification system is developed [5, 6]. The Dana Point expert group has published a consensus of PH classification based on pathology, survival, natural history/epidemiology, etiology, and response to the treatment [5]. Among them, one of the most classical types is pulmonary arterial hypertension (PAH). PAH specifies that the disease primarily restricted to the pulmonary arterioles, a typical characteristic which shows an elevated pulmonary arterial pressure [2, 3]. The pathological consequence of PAH is the structural remodeling of pulmonary arteries (PA), where increased proliferation of pulmonary artery smooth muscle cells (PASMC) and dysfunction of pulmonary artery endothelial cells (PAEC) occur in the vascular bed [7–9]. The morphological changes consist of hypertrophy of the tunica media, multicellular vascular lesions which obstruct and obliterate pulmonary arterioles leading to intimal thickening. The obstructed vessels limit the blood flow* via* PA and increase right ventricular afterload leading to right ventricular hypertrophy (RVH) and RV dysfunction [10–12]. At molecular level, it is believed that the remodeling events in PH demand the participation of all cell-types present in the pulmonary arteries and that influence the pathological manifestation in the pulmonary vessel wall. The contributing factors that influence the remodeling process are hypoxic state, inflammation, vessel injury, and oxidative stress in the pulmonary vessels. As all forms of PH have in common an altered production of various endothelial vasoactive mediators, such as nitric oxide, prostacyclin, or endothelin- (ET-) 1, to establish the correct balance between vasoconstriction and vasodilatation [13–16]. Currently, the management for PH is aimed at optimizing cardiopulmonary interactions by targeting prostacyclin, endothelin, and nitric oxide signaling pathways [17]. The most commonly used treatment regimen of PH is the use of prostacyclin analogues (Alprostadil, Epoprostenol, Treprostinil, and Iloprost), endothelin receptor antagonists (Bosentan, Ambrisentan), and inhaled NO. In addition, phosphodiesterases (PDEs) inhibitors; PDE-3 inhibitors (e.g., Milrinone and Enoximone), and PDE-5 inhibitors (e.g., Sildenafil and Tadalafil) are used to treat PH. They were used as an alternative therapeutic strategy which targets downstream components of the NO signaling pathway by inhibiting PDE-5, the enzyme that catalyzes the conversion of cGMP to GMP. Despite the advancement of modern surgery or PH-specific therapy, the mortality of PH patients still remains high, ranging between 22.2% and 54.5% [18].

Although PH (or PAH) is well-studied encompassing both cardiac and vascular boundaries, the precise cellular and molecular mechanism of initiation and progression of PH are not completely understood and are still being explored. There is no cure of this disease and current therapies are limited to reverse the vascular remodeling. Evolving evidence indicates that dysregulation of microRNAs (miRNA or miR) contributes to PH pathogenesis [19–24]. Indeed, an emerging body of evidence demonstrates that a fine balance in miRNA levels seems to be a fundamental to maintaining homeostasis in the pulmonary vasculature and an imbalance with miRNA level playing a critical role in the pathogenesis of PH by regulating a set of targeted genes [25]. This review will collate vascular remodeling during PH, miRNA biogenesis, recent advances on miRNA modulation in PH, therapeutic opportunity, and conclusion.

## 2. miRNA Biogenesis

**Figure 1:**
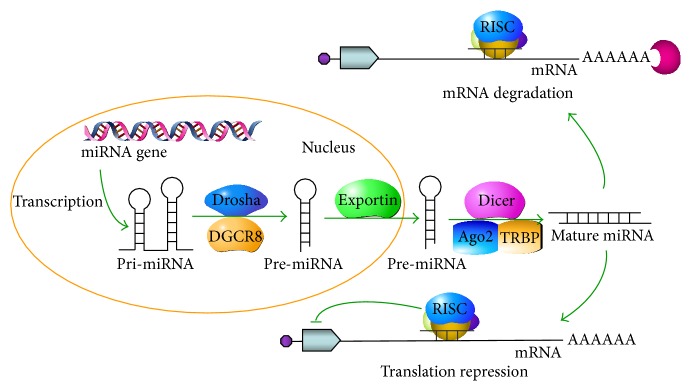
miRNA biogenesis. The miRNAs are transcribed by RNA polymerase II as primary transcript of miRNA (pri-miRNA). The pri-miRNA is the cleaved by RNase III enzyme, Drosha, along with several cofactors including DGCR8 and produces the stem-loop precursor miRNA (pre-miRNA). The pre-miRNA is then exported out of the nucleus by Exportin-5 to the cytoplasm. In the cytoplasm, the pre-miRNA is diced-up by Dicer resulting miRNA duplex, ~22 nucleotides long. The mature miRNA is incorporated into the RNA-induced silencing complex (RISC) which contains Argonaute (Ago) and is guided to the 3′-UTR of target mRNAs. The gene silencing is achieved by either mRNA degradation or translational repression.

The miRNA(s) are composed of a vast family of short, noncoding RNAs (~22 nucleotide long). miRNAs are found from a single cell organism to plant and higher animals and even viruses [37–39]. In humans, more than 2,500 miRNAs have been reported in miRBase 20.0 database [40]. The biogenesis (canonical) pathway of mammalian miRNAs is a two-step enzymatic process initiated in the nucleus and then transported to cytoplasm (Figure 1). The transcription of miRNAs generally processed by RNA polymerase II (less frequently by RNA polymerase III) and are typically capped and polyadenylated [41, 42]. As a result, a long primary miRNA transcripts (pri-miRNA) containing a stem-loop structure is developed which is recognized by a large protein complex, called the Microprocessor, the main components of which are the RNase III Drosha and DiGeorge Syndrome Critical Region 8 (DGCR8) [43–48] (Figure 1). For most pri-miRNA, Drosha is contributing the cleavage process called “cropping” with the assistance of its binding partner protein, DGCR8 [41, 48, 49]. The resultant product is a ~60 nucleotides long, hairpin-structured, called precursor miRNA (pre-miRNA). DGCR8 directly interacts with the pri-miRNA stem and flanking segments, a crucial measurement for one end of the mature miRNA [45]. In addition to the canonical pathway of miRNA biogenesis described above, an alternative pathway also exists that are independent of Drosha. In alternative pathways, the miRNA can be released as mitrons (pre-miRNA like introns) [50] from pri-miRNA and proceed for miRNA processing unit without assistance of Microprocessor [50, 51].

The pre-miRNAs are actively transported from the nucleus into the cytoplasm by Exportin-5 (Exp-5) [52] (Figure 1). In the cytoplasm, another RNase III enzyme, called Dicer, catalyzing the process with the assistance of partner molecules Argonaute (Ago2), HIV-1 transactivation response RNA-binding protein (TRBP) and/or Protein activator of PKR kinase (PACT), producing a short dsRNA duplex, miRNA/miRNA ^*^ of approximately 22-nucleotide length [44, 53–56]. The miRNA/miRNA ^*^ duplexes are then incorporated into a ribonucleoprotein (RNP) complex—called miRISC (RNA-induced silencing complex)—that plays a critical role in the miRNA-mediated mechanism of gene regulation. During assembly process of the miRISC, the miRNA/miRNA ^*^ duplex is loaded into the Ago, and the strands (called the “passenger strand”) are released and degraded [57]. Eventually, the bound miRNA strand (called the “guide strand”) dictates miRISC to interact with partially complementary sequences in target transcripts (localized within the 3′UTR) and primarily triggers mRNA deadenylation and degradation [58].

## 3. Key miRNA in the Pathogenesis of PH

MicroRNAs (miRNAs) are small, endogenously expressed noncoding RNAs that regulate gene expression at posttranscriptional level, via degradation or translational inhibition of their target mRNAs [37, 59]. miRNAs are ~22 nucleotides in length which bind to the 3′ untranslated region of specific target genes and thereby suppress/inhibit the translation of target genes [60, 61]. miRNAs are key regulators of a wide range of cellular processes and play a pivotal role in vascular inflammation and cardiovascular pathologies inclusive of PH. From extensive studies from the past few years, it has become apparent that miRNAs are expressed in a cell- and tissue-specific manner and are critically involved in various biological processes. The degree of imbalances in their expression may lead to various diseases. Emerging evidence indicates that miRNAs contribute an important role in the maintenance of pulmonary vascular homeostasis and in the pathogenesis of PH [62]. In the following part, a discussion of the roles of miRNAs in PH-related signaling pathways is provided.

### 3.1. miR-21

miR-21 is a ubiquitously expressed miRNA that is traditionally considered to be an oncogenic miRNA (oncomiR). The two-channel microarray was performed to quantify miRNA expression in whole lung extracts during the development of PH or PAH caused by chronic hypoxia or monocrotaline in rats. It was suggested that miR-21 expression was downregulated in MCT-induced PH model, but not in chronic hypoxia rats. The similar change of miR-21 was observed in cultured PASMCs and PAF. miR-21 showed a similar expression level in both normoxic hypoxic cells, whereas TGF-*β*1, an important regulator of pulmonary vascular remodeling in PH, reduced expression of miR-21. This suggests that although hypoxia- and monocrotaline-induced PH shares some common cellular processes driving the characteristic vascular remodeling, the different pathobiology induced by hypoxia and monocrotaline may lead to the different regulation of miR-21 expression. Furthermore, the downregulation of miR-21 was confirmed in human lung tissue and serum from patients with idiopathic PH [20]. Since BMPs induce smooth muscle cell differentiation through upregulating the expression of miR-21, the downregulation of miR-21 in this setting may relate to the reduced BMP signaling and contribute to the alteration of smooth muscle cell phenotype in PH [63].

However, Yang and colleagues reported that miR-21 expression was increased in distal small pulmonary arteries of hypoxia-exposed PAH mice and levels of BMPR2, WWP1, SATB1, and YOD1, the putative miR-21 targets, were decreased in the same tissue. Transfection of miR-21 mimics also led to the reduced expression of BMPR2, SATB1, and YOD1 in PASMCs [23]. The crucial role of miR-21 in vascular pathology has been evidenced by the results that the blockade of miR-21 impeded the development of intimal hyperplasia after acute vascular injury and bleomycin-induced pulmonary fibrosis [28, 64]. Yang et al. also found that inhibition of miR-21 alleviated chronic hypoxia-induced PH and attenuated pulmonary vascular remodeling. In human PASMCs, overexpression of miR-21 promoted, whereas sequestration of miR-21 abrogated cell proliferation and the expression of cell proliferation-associated proteins. The miR-21 null mice showed an exaggerated PH response to hypoxia, suggesting a major role of miR-21 in the pathogenesis of chronic hypoxia-induced pulmonary vascular remodeling and PH [23]. Although an association between miR-21 and PH (PAH) is identified, the function of miR-21 in the development of PH is inconsistent in many experimental models. Therefore, further investigations are required to clarify the role of miR-21 in the pathogenesis of PH.

### 3.2. miR-204

The miR-204 appears to be the first miRNA that showed a mechanistic link between pulmonary arterial remodeling and cellular function. miR-204 was reported to be decreased in rodent lungs with hypoxia- and MCT-induced PAH and lung specimens from patients with PAH [65]. The reports suggested that STAT3 activation contributed a crucial role in regulating miR-204 in PASMC. It was further demonstrated that miR-204 inhibition increased expression of SHP2, triggered the activation of Src kinase, STAT3, and nuclear factor of activated T cells (NFAT), and thereby reduced apoptosis and was promoted proliferation of PASMCs. Finally, delivery of synthetic miR-204 mimic to the lungs lowered pulmonary artery pressure, reduced medial wall thickness, normalized levels of miR-204, SHP2, and STAT3, and alleviated the disease severity [65]. This study may indicate the safe and effective use of mimic delivery to the pulmonary vasculature for future therapeutic purpose. Interestingly, Wei et al. have identified reduced level of miR-204 in the buffy coat of human subjects may correlate with PAH severity and might serve as a circulatory biomarker for PH [66].

### 3.3. miR-143/145 Cluster

The miR-143/145 cluster was considered to be expressed specifically in vascular smooth muscle cells [67]. Expression of miR-143/145 was driven by TGF-*β* and BMP4 and induced contractile gene expression through downregulating KLF4 and myocardin [31, 68, 69]. The elevated levels of miR-145 were observed in primary PASMCs cultured from patients with BMPR2 mutations and also in the lungs of BMPR2-deficient mice, suggesting a role of BMPR2 signaling in modulating miR-145 expression [24]. Caruso and colleagues also found that miR-145 was downregulated in patient samples obtained from idiopathic and congenital PH but upregulated in plexiform lesions. The role of miR-145 in PAH was further explored and elevated expression of miR-145 was shown in the wild type mice exposed to hypoxia. miR-145 deficiency and a locked nucleic acid anti-miR-145 resulting in significant protection from hypoxia-induced PH may represent a potential therapeutic target [24].

### 3.4. miR-17-92 Cluster

The miR-17-92 cluster encodes seven related miRNAs, which result from the transcription of a single pre-miRNA and is further processed and cleaved to the mature miRNAs [70]. miR-17-92 cluster was retrieved as potential modulators of BMPR2 signaling by performing a computational algorithm on the BMPR2 gene. Overexpression of miR-17-92 resulted in a marked reduction of BMPR2 protein level, and BMPR2 was proved to be directly targeted by miR-17 and miR-20a by using a BMPR2 reporter in HEK293 cells. STAT3 was found to be involved in IL-6 signaling-mediated upregulation of miR-17-92 cluster and the subsequent downregulation of BMPR2, since a highly conserved STAT3-binding site exists in the promoter region of miR-17-92 gene [71].

The role of a cholesterol-modified antagomir to miR-20a in hypoxia-induced PH was explored by Brock et al. miR-20a restored functional BMPR2 signaling in human PASMCs and intraperitoneal administration of anti-miR-20a increased BMPR2 levels and alleviated vascular remodeling in lung tissue of hypoxic mice [72]. Antagomirs to miR-17 and miR-92a reduced muscularization of pulmonary arteries in the hypoxic mouse and monocrotaline rat models of PH, but only anti-miR-17 decreased RVSP and parameters of right heart dysfunction [22].

### 3.5. miR-130/301

Bertero and colleagues identified miR-130/301 family as a master regulator of cellular proliferation in PH by constructing* in silico* a network of genes and interactions based on curated seed genes with known importance in PH. miR-130/301 expression was found to be increased in lungs of mice suffering from PH induced by SU5416 administration with chronic hypoxia, in lungs of rats with MCT-induced PH and in lungs of juvenile sheep with shunt-induced PH. In both human PAECs and PASMCs, multiple PH inducers, including hypoxia, IL-1*β*, and IL-6, increased miR-130/301 expression and hypoxia was found to upregulate miR-130/301* via* a dependence on HIF-2*α* and POU5F1/OCT4. It was further validated that miR-130/301 modulated apelin-miR-424/503-FGF2 signaling in PAECs and miR-130/301-PPAR-*γ* axis controlled proliferation of PASMCs by increasing STAT3 expression and activity and repressing miR-204 expression. In hypoxia-induced PH mouse model, induction of miR-130/301 was promoted while miR-130/301 inhibition prevented PH pathogenesis [73].

## 4. miRNA in PASMCs Plasticity in PH

PH is broadly considered to be a vascular disease as vasoconstriction in the pulmonary artery is a prime cause for the development of PH. The hyperproliferation of pulmonary vascular cells (mainly VSMC) and subsequent neointima formation in the small PAs are hallmark in PH. On the other hand, pulmonary arterial endothelial cells (PAECs) further contribute a critical role in vascular homeostatic balance in the pulmonary vascular bed by orchestrating the vessel tone, leucocytes trafficking, and so forth [74, 75]. Another cell type called adventitial fibroblasts also participate a critical role in PH [76]. Proliferation of fibroblasts is reported in hypoxia-induced PH that plays a role in adventitial thickening [77, 78]. Under pathological situation like PH (PAH), the endothelium becomes dysfunctional which allows the penetration of infiltrating molecules, increased leucocytes adhesion, getting resistant to apoptosis that result in severe remodeling in the pulmonary vessels. These infiltrating molecules invade the barrier of neighboring cells like smooth muscle cells and activating the resident of adventitious fibroblasts. The remodeling process is basically an interaction between these cell-types present in the pulmonary arterial layers that resulting in a marked histological change in the pulmonary vasculature [75]. An emerging body of evidences suggests that the miRNAs play a pivotal role in the vascular cell integrity and plasticity during the progression of PH. Here, we assemble those miRNAs that participate in vascular remodeling in PH.

Although identification of miRNAs in vascular diseases like PH is relatively new, but the earliest association connecting VSMC remodeling with miRNAs was reported in 2007. Ji et al. described the miRNA signature in the vascular wall of balloon-injured carotid artery rat model [64]. They showed that miR-21 was upregulated and the cellular effects were targeted by PTEN and Bcl_2_ for neointimal lesion formation [64]. The initial studies of miR-21 in vascular remodeling were followed by series of milestone works that include miR-143/145 cluster [24, 31, 79–83]. Two of the key miRNAs involved in regulation of the phenotype of SMCs are miR-143 and miR-145. miR-145 is transcribed bicistronically along with miR-143 from human chromosome 5 [24]. With relevance to PAH, downregulation of miR-145 is associated with increased proliferation of neointimal cells [31]. Downregulation of target genes klf4 and klf5 by activation of miR-143/miR-145 upregulates SMC-specific genes such as SMA, calponin, and SM22-*α*, triggering differentiation and lowering the proliferation rates in vascular SMCs [31, 82]. Another miRNA, miR-221/222 that triggers PDGF signaling in VSMC is thought to contribute in neointimal proliferation [35, 84, 85]. It is demonstrated that inhibition of miR-221 prevented PDGF induced proliferation, while forced expression of miR-221 increased proliferation and reduced the expression of VSMC marker [35, 84].

In addition to the above miRNAs, other miRNAs are reported to regulate SMC gene expression in hypoxia-induced PH. They included miR-210, miR-124, miR-204, and miR-138. It is demonstrated that miR-210 that acts as a hypoxia-inducible miRNA both* in vitro* and* in vivo*, inhibits PASMC apoptosis in hypoxia by specifically repressing E2F3 [86]. A cell-based high throughput screening of a human miRNA library with the NFAT luciferase reporter system identified miR-124 as a new candidate in PH. It showed decreased NFAT reporter activity and, decreased dephosphorylation and the nuclear translocation of NFAT [87]. An elegant study by Courboulin et al. demonstrated that miR-204 downregulation correlates with PH severity and responsible for proliferative and antiapoptotic phenotypes of PH-PASMCs targeting STAT3 and NFATc [65]. Interestingly, a recent study showed that a serine/threonine kinase Mst1, a modulator of cell death, appears to be a target of miR-138 [88]. The authors suggest that miR-138 may be a negative regulator of PASMC apoptosis in hypoxic mediated PAH. A panel of miRNAs which are shown to modulate SMC fate and modulation are listed in Table 1 [26–36].

## 5. miRNA as Therapeutic Target for PH

Therapeutic target which defines the treatment of a disease by means of a well-defined biological molecule. In this regard, miRNAs, a “tiny” RNA molecule, have become an important gene modulator of various biochemical, physiological, and cellular functions. As we observed that either deficiency or abundance of a specific miRNA or cluster of miRNAs contributed a pathological state of many cardiovascular diseases including PH, it is reasonable to accept those miRNAs as therapeutic target for diagnosis and therapeutic intervention. This is because we can utilize ability of a single miRNA to control the expression of multiple (hundreds) proteins suggest that changes of a single miRNA may influence several signaling pathways associated with pathological disease state. While each target is regulated subtly, the additive effect of coordinated regulation of a large suite of transcripts is believed to result in strong phenotypic outputs. Several animal models in diverse disease pathology showed usefulness of miRNAs as target molecules for therapeutic benefit; this review will focus on vascular remodeling associated with PH. At therapeutic strategic stand-point, the aberrant miRNA expression can be modulated or restored to normal by two main approaches: an anti-miRNA (anti-miR) and miRNA mimic. The former can be applicable to those miRNAs whose expressions were increased in disease pathology and, therefore, silencing or inhibiting will be beneficial. To modulate miRNA expression, the current strategy utilized chemically modified, cholesterol-conjugated single-stranded RNA analogues complementary to the mature miRNAs for antagomir or mimics, respectively [89]. Synthetic modified oligonucleotides are currently used as potential “antagomirs” for miRNA silencing [90]. An elegant work demonstrated by Liu et al. in a balloon-injury model represents a successful knocking down of miR-221/222 that suppressed VSMC proliferation and neointimal lesion formation [35]. Recently, Brock et al. have shown that treatment with antagomiR-20a restores the levels of BMPR2 in pulmonary arteries and prevents vascular remodeling in hypoxia-induced PAH [72]. For downregulated miRNAs, a mimic approach is generally undertaken which basically rescued the underexpressed miRNA in the tissue under pathological situation [89]. This strategy is sometimes referred to as miRNA replacement as it reintroduced the similar depleted miRNA those were downregulated during diseases progression. Therefore, by introducing the miRNA “mimic,” the cells can restore the function as the miRNA “mimic” is expected to target the same set of mRNAs that is also regulated by the endogenous miRNA. In a very comprehensive study by Courboulin et al., they demonstrated that reestablishing of miR-204 level by delivery of synthetic miR-204 into the lungs of experimentally-induced PAH animals significantly reduced the disease pathology [65].

Although the miRNA-based intervention is attractive and seems to be feasible, however, several challenges are noted. First, the critical miRNAs responsible for PH must be confirmed definitively in well-accepted animal models along with explanted human PAH samples, biopsies, and so forth, second, the cellular, molecular and physiological function of these miRNAs should be performed in diseases progression and prevention; third, the delivery route should be precisely targeted or restricted to lung vascular cells, for example, PAEC, PASMC, or fibroblasts. This will minimize the off-target effects on the neighboring cells. Finally, the dose of antagomir or mimic delivery to the vascular bed should be carefully monitored further to avoid off-target effects. Therefore, our future direction should be focus of vascular cell-specific delivery of miRNA mimic or inhibitors. Additionally, a regulated release of miRNAs by conjugating nanoparticle will be considered for long-term use. Finally, miRNA-mediated therapeutics can be achieved by inhalation which may reduce the potential off-target issues to the other organs.

## 6. Can Circulatory miRNA Be Used as a Biomarker in PH?

Biomarker generally refers to measurable substance in biological state. At clinical stand-point, it is extremely important as it predicts the “medical” outcome of a disease and its progression. Over the past few years, it has been demonstrated that miRNAs are found in the blood, plasma, urine, platelets, and saliva in a surprisingly stable form [91–97]. The stability of miRNA in the extracellular environment offers a great opportunity to consider as “biomarker” at clinical settings. Using miRNA as a “biomarker” offers many advantages like early prediction of diseases, differential expression during disease progression/pathologies, high degree of specificity, and sensitivity and importantly having longer half-life in the system. Accumulating evidence suggested that the stability of miRNA in extracellular environment illustrated by packed with lipid vesicles, wrapped in protein or lipoprotein complexes [98–100]. In the context of cardiac diseases that include myocardial infarction, hypertrophy and heart failure; several miRNAs are identified and predicted to be considered as “biomarker” [101–104].

The miRNAs are circulating freely in the mammalian blood and can be predicted as “biomarker” for early diagnosis of cardiovascular diseases in humans [102, 105–108]. Several evidences indicate that miRNAs are secreted as micro vesicles or exosome and apoptotic bodies that may be responsible for release the miRNAs into the circulation [109–112] and are extremely stable in the blood or serum [91]. Rhodes and colleagues measured plasma levels of miRNAs in eight patients newly diagnosed with PAH and eight healthy controls by use of microarray analysis. Fifty-eight miRNAs showed differences in plasma concentration between the two groups and miR-150 was largest downregulated in PAH. Reduced expression of miR-150 predicted 2-year survival and correlated with disease severity. The miR-150 level was also found to be significantly reduced in circulating microvesicles and lymphocytes from patients with PAH [113]. Courboulin et al. reported that downregulation of miR-204 in buffy-coat cells correlated with PAH severity and might serve as a circulatory biomarker of PAH [65]. On the contrary, increasing higher plasma levels of miR-130/301 family members were observed in patients with increasing hemodynamic severity of PH [73].

Our previous study detected the pattern of miRNAs in buffy-coat samples from mild-to-severe human PH subjects compared with the control subjects by miRNA array. Our study revealed that moderate to severe PH in human subjects are associated with significant downregulation of plasma levels of circulatory miR-1, miR-26a, and miR-29c. The kinetics of the changes differed from moderate to severe PH human subjects as we observed further downregulation of the above miRNAs in severe PH. We also found a set of upregulated miRNA in the circulation of PH subjects. miR-21, miR-23b, miR-130a, miR-491, and miR-1246 were moderately upregulated in moderate PH subjects and were more pronounced in severe PH. Another set of miRNAs that include miR-133b, miR-204, and miR-208b were significantly upregulated in all moderate PH subjects and showed further increment in severe PH subjects [66]. Our study provided the evidence for the first time that circulating miRNAs in the setting of PH may be used as early detection parameters for PH. Nevertheless, the data presented were based on limited study population and the results need to be confirmed in a larger cohort study. Furthermore, the dysregulation miRNA in PH remains to be tested whether these circulatory miRNAs have any influence in the target genes.

Circulatory miRNAs are considered to be novel promising biomarkers for detection of PH. However, at current stage, using circulatory miRNA as diagnostic tool for detecting PH is still in its infancy. The key advantage of circulating miRNAs is a noninvasive testing procedure and relies on the stability of miRNAs in the circulation and their storage condition. We give credit to the concept of quick determination of secretory molecules (miRNAs) in the blood; but the importance of miRNA must be reevaluated at the clinical setting or model at multiple laboratories. Another limitation is the small number of cohort study which appears to be heterogeneous clinical sample-type. These points may consider as preliminary-type of observation and need further confirmatory studies for considering miRNAs as biomarkers for clinical use.

## 7. Conclusion

Understanding of miRNAs role and function in the pulmonary circulation will offer and contribute a great potential to the pathogenesis of PH or PAH. The miRNA signature can be used as a diagnostic tool for the development of therapeutic strategy. The characterization of these miRNAs under various experimental settings (*in vitro* and* in vivo*) should be performed along with the human subjects. The outcome will validate both physiological and pathological roles of these miRNAs during the development and progression of PH prior to consider them for therapeutic intervention or future clinical trials. Furthermore, use of disease specific mouse models, conditional transgenic, and knockouts are necessary to elucidate the functional role* in vivo* and verify its therapeutic potential, both of which remain largely unexplored. More novel approaches such as combination of Argonaute protein immunoprecipitation, deep sequencing, or proteomic profiling approaches are required to identify its potential* bona fide* targets responsible for its functional impact.

## Figures and Tables

**Table 1:** Vascular smooth muscle cells and miRNA modulation.

miRNA	Function	Target gene(s)	Reference
miR-1	Promote VSMC differentiation	Myocardin, KLF4	[26, 27]
miR-21	Promote VSMC differentiation	PDCD4	[28]
miR-24	VSMC proliferation and repression of contractile gene expression	Tribbles-like protein 3, SMURF1	[29]
miR-26a	Promote VSMC dedifferentiation	Smad1 and Smad4	[30]
miR-143/145	Promote VSMC differentiation	KLF4, KLF5, ELK1, versican, BMP4, MRTF	[31–33]
miR-146	Promote VSMC dedifferentiation and proliferation	KLF4	[34]
miR-221/222	Promote VSMC dedifferentiation and proliferation	p27Kip, c-kit	[35, 36]
